# BioPhysConnectoR: Connecting Sequence Information and Biophysical Models

**DOI:** 10.1186/1471-2105-11-199

**Published:** 2010-04-22

**Authors:** Franziska Hoffgaard, Philipp Weil, Kay Hamacher

**Affiliations:** 1 Theoretical Biology and Bioinformatics, Institute of Microbiology and Genetics, Department of Biology, TU Darmstadt, Schnittspahnstrasse 10, 64289 Darmstadt, Germany

## Abstract

**Background:**

One of the most challenging aspects of biomolecular systems is the understanding of the coevolution in and among the molecule(s).

A complete, theoretical picture of the selective advantage, and thus a functional annotation, of (co-)mutations is still lacking. Using sequence-based and information theoretical inspired methods we can identify coevolving residues in proteins without understanding the underlying biophysical properties giving rise to such coevolutionary dynamics. Detailed (atomistic) simulations are prohibitively expensive. At the same time reduced molecular models are an efficient way to determine the reduced dynamics around the native state. The combination of sequence based approaches with such reduced models is therefore a promising approach to annotate evolutionary sequence changes.

**Results:**

With the R package BioPhysConnectoR we provide a framework to connect the information theoretical domain of biomolecular sequences to biophysical properties of the encoded molecules - derived from reduced molecular models. To this end we have integrated several fragmented ideas into one single package ready to be used in connection with additional statistical routines in R. Additionally, the package leverages the power of modern multi-core architectures to reduce turn-around times in evolutionary and biomolecular design studies. Our package is a first step to achieve the above mentioned annotation of coevolution by reduced dynamics around the native state of proteins.

**Conclusions:**

BioPhysConnectoR is implemented as an R package and distributed under GPL 2 license. It allows for efficient and perfectly parallelized functional annotation of coevolution found at the sequence level.

## Background

One of the biggest challenges in the post-genome era [1] is to understand how proteins evolve, fold, and structurally encode their function. Understanding the underlying coupling of protein sequence evolution and bio-mechanics is the first step to develop new drugs and annotate molecular evolution in physical space. Exploring the accessible sequence space of a protein provides insights into its evolutionary history and phylogenetic relations. Mutual information (MI), an information-theoretical approach, is widely used to detect coevolution [2-9] at the sequence level within a protein or among several molecules. Such statistical methods allow high-throughput investigations, but the biophysical/-chemical implications of protein sequence changes are not revealed by these methods.

In general a sequence change is fixated in molecular evolution, if it has proven to be useful in the physical realm by benefitial biophysical properties and functions. Interactions between proteins as well as functional aspects of monomers are largely conserved throughout evolution, which implies coevolution among residues. Such coevolution contributes to maintain crucial interactions between these coevolving residues. To explore the physical realm, molecular dynamics (MD) simulations and related methods are routinely employed. Their applicability is restricted to just a few mutants due to severe computational demands of MD. To overcome this drawback a number of coarse-grained models have been developed in recent years [10-12]. In contrast to MD simulations, these models allow high-throughput screening of natural and unnatural mutations.

Hamacher [13] developed a protocol to integrate both the information from sequence-driven methods and the mechanical aspects derived by biophysical interaction theories, eventually bridging the gap between statistical bioinformatics and molecular dynamics/biophysics. Connecting both points of view proved to be essential for the construction of molecular interaction networks [12] and helps to understand thermodynamical properties and evolutionary changes [14]. The purpose of BioPhysConnectoR is to provide evolutionary biologists and other bioinformatics researchers with these protocols and allow for future development of new protocols to integrate information space and physical space in a holistic picture of molecular evolution.

## Implementation

The BioPhysConnectoR package is an add-on package to the statistical software R version 2.8+ [15]. BioPhysConnectoR includes source code from the bio3d[16] package and uses functions from the matrixcalc[17] and the snow[18] package. To address runtime features we integrated native C/C++-routines for more complex computational tasks that are callable from within the R environment. We provide low-level routines to account for specific tasks as well as high-level routines to process complete protocols. These can be customized via various arguments. BioPhysConnectoR includes utilities to perform the following tasks:

1. An alignment given in fasta format can be read and information theoretical measures such as MI and entropy can be computed. It is possible to compute a null model [19] to estimate the statistical relevance of the derived MI values.

2. It is possible to read a pdb file and compute the Hessian as well as the covariance matrix for a coarse-grained anisotropic network model (ANM) [10,11], thus computing reduced dynamical properties of the molecule. This is done in the ANM in a harmonic approximation of the full, atomistic potential. The actual computation is performed by a singular value decomposition (SVD). Additionally B-factors can be extracted from the covariance matrix.

3. *In silico *experiments can be performed by changing the underlying protein sequence or "breaking" amino acid contacts for the computation of biophysical properties. For given alignments, the outcome can be combined with the respective MI or joint entropy values.

4. The self-consistent pair contact probability (SCPCP) [20] method is included as an additional method to derive B-factors and further biophysical properties from a coarse-grained approach.

5. Some additional matrix routines are implemented.

## Methods

### Information-theoretical approach

As a measure for coevolution among residues we use MI [2-9], defined as [21]:(1)

where *x *and *y *are realizations of the random variables *X*_*i *_and *Y*_*j *_drawn from a set , taken from a multiple sequence alignment as columns *i *and *j *- resulting in an MI matrix (MI_*ij*_). For proteins we are concerned with the symbol set of the 20 standard amino acids _*AA*_, which can be expanded to include the gap character and an extra character for non-standard amino acids . The probabilities *p*_*i*_(*x*), *p*_*j*_(*y*), and *p*_*ij*_(*x*, *y*) are obtained as the relative frequencies of amino acids within the columns of a multiple sequence alignment.

### Biophysical approach

Reduced molecular models [10,11] are obtained by using only a coarse-grained representation of amino acids, such that each amino acid is represented by a bead at the center of its respective C_*α *_atom.

Interactions between amino acids in contact with one another are modeled as harmonic springs, with one spring constant, *K*, weighting the strength of interactions between adjacent amino acids in the sequence (*i *and *i *+ 1), and individual "sequence-dependent" spring constants, *κ*_*ij*_, controlling other interactions. The total potential for a protein in any conformation is thus(2)

where *s*_*i*, *i*+1 _is the distance of the C_*α *_atoms at adjacent positions (i.e. covalently attached pairs) at a time point in a test conformation, and  is the distance of the same atoms in the native structure. *C *contains all pairs of residue positions *i *and *j *with non-covalent contacts that are within a given cutoff. The amino acid-specific statistical contact potential matrices of Miyazawa and Jernigan (MJ) [22] and Keskin et. al. (KE) [23] were used for the non-covalent spring constants, *κ*_*ij *_to provide for sequence specificity [11]. Using MJ and KE, the ANM was shown to improve the correspondence to experimental results [11,12]. Other weighting schemes for amino acids contacts can be provided by the user as arguments to the respective function in BioPhysConnectoR.

For the investigation of the mechanics of the molecule, we construct the Hessian matrix of the potential *V*. Via SVD we compute the eigenmodes and -frequencies to derive the covariance matrix *M*. The entry  reads:(3)

for *α*, *β *= x, y, z. The eigenvalues of the Hessian are denoted with *λ*_*k *_and the respective eigenvectors with . *i*, *j *are the indices of the residues.

*M *includes all mechano-dynamical information obtainable by the model in eq. 2. The covariance matrix turns out to be the inverse of the Hessian in this model. Considering three translational and three rotational degrees of freedom, the sum over *k *leaves out the first six eigenvalues that vanish. Thus we effectively compute the Moore-Penrose pseudo-inverse [24,25]. The isotropic B-factors can directly be derived from this matrix as(4)

Introducing mutations leads to changes in the physical realm and thus to a covariance matrix *M*^mut ^different from the "wild-type" one *M*^wt^. The impact of a specific mutation on the biomechanical behavior of the molecule can be estimated determining the Frobenius norm (FN) of the respective covariance matrices as follows(5)

Such elastic network models were extended to include thermodynamics - including phase transitions indicating folding/unfolding events. The extension we implemented is the SCPCP approximation first proposed by Micheletti *et al*. [20] and later used by Hamacher *et al*. [12] to investigate binding free energies of ribosomal subunits. The SCPCP can produce non-harmonic effects beyond properties one usually would expect in simple models. In particular it can show finite-size equivalents of "phase transitions", e.g. protein unfolding.

## Results and Discussion

**Figure 1:**
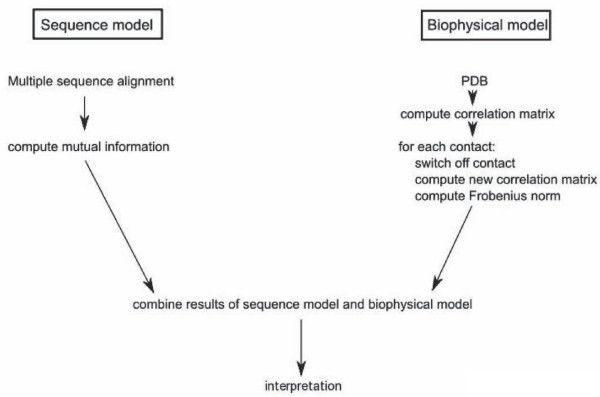
**Flowchart**. The protocol describes the two major workflows the package provides to combine results from investigations concerning sequence and structure of a molecule. Key positions for coevolution and function can be identified using a combination of the workflows.

**Figure 2:**
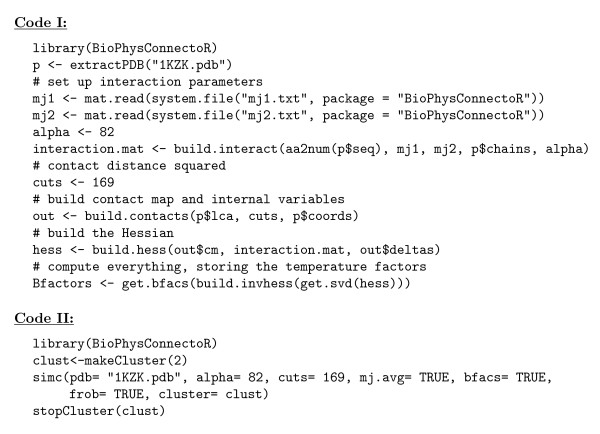
**Example code**. At the top (Code I), a detailed example for the computation of the B-factors using the provided low-level routines is shown. At the bottom (Code II), the function call of simc is shown with all parameters given to the function to compute the values of Figure 4. The parameter alpha represents the strength of peptide bonds and is set to 82 per default, the parameter cuts denoting the squared cutoff distance for a contact has a default value of 169. If no interaction weighting matrix is given, the MJ and KE matrices [22,23] are used to describe the specific intra- and interchain interaction strengths, respectively, eventually averaged if mj.avg = TRUE. If the parameter bfacs is set TRUE, for each broken contact the computed B-factors are written into files. Frobenius norms are only computed and returned if frob = TRUE. With the parameter cluster it is possible to provide an initialized cluster for a parallel computation.

In this section we present an example application of the BioPhysConnectoR package to the HIV-1 protease [PDB:1KZK]. The molecule is a homo-dimer with 99 amino acids in each chain. We show the work flow of the employed protocol in Figure 1. To illustrate the usage of the BioPhysConnectoR routines we provide a code example (see Figure 2) as well. The pdb file serves as input for the computation of the biophysical properties. To gain insight into evolutionary features, we use data provided by Chen *et al*. [26]. The nucleotide sequences were translated into amino acid sequences. As we are concerned with about 45160 sequences, we do not need to consider finite size effects [19] in the MI results.

The alignment is read and MI values are computed. We then pick those residue pairs with the highest MI values that are non-covalently in contact within the cutoff of 13Å. The pdb is read and the C_*α *_atoms of the first chain are selected. We compute the covariance matrix *M*^wt ^for this system. Afterwards we "break" the contact for each previously selected amino acid pair (*a*, *b*), one at a time, and compute a respective new covariance matrix *M*^mut, (*a*, *b*)^. The corresponding change in the mechanical behavior can be annotated by the Frobenius norm *f *(see eq. 5) between these two matrices.

**Figure 3:**
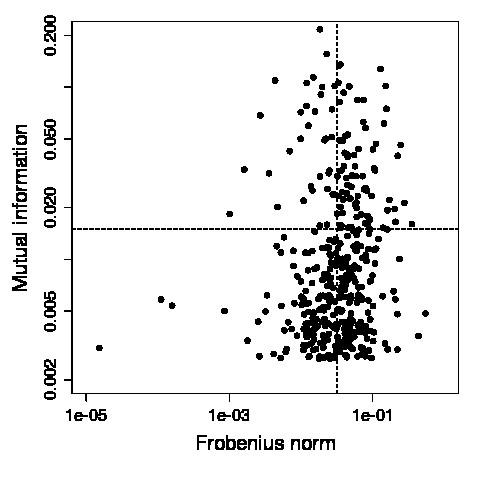
**MI vs. *f***. We applied the protocol proposed by Hamacher [13] to a monomer of the HIV-1 protease [PDB:1KZK]. First, we identified coevolving positions of the protein sequences using MI. We then switched off the 400 contacts with the highest MI values consecutively and computed the Frobenius norm to determine the impact of mechanical changes. Plotting the Frobenius norm against MI helps to identify key residues. Interactions between those are subject to a high selective pressure (indicated by high MI) and have the largest influence on the protein dynamics and stability (large Frobenius norm *f*).

We plotted the MI value of each residue pair (*a*, *b*) against the Frobenius norm *f*^(*a*, *b*) ^when breaking this specific contact in Figure 3. The figure shows the separation into four cases as discussed in [13]. We classify entries by the proximity to the four points located at angles 45°, 135°, 225°, 315° respectively. Pairs with low MI values have undergone little coevolution and thus coevolve less than those with higher values. If these pairs show also small FN scores, their contact has no relevant meaning. The low coevolution can be explained by the rather unimportant impact the interaction of such a pair has on the overall molecular dynamics. On the other hand, high FN values indicate large changes in the covariance matrix within the modeling framework we implemented. These changes in the covariance matrix suggest in turn relevant changes in the mechanics of the molecule when "breaking" this specific contact. Details of this protocol can be found in [13]. Note that our protocol allows the annotation of the non-correlating MI-FN-value pairs: usually one would not expect correlation. Instead one is interested why particular high MI appears for pairings. One out of several biophysical aspects stems from the dynamics of around the native state - computable by BioPhysConnectoR. Additional effects might include e.g. electrostatics or binding partner recognition capabilities.

**Figure 4:**
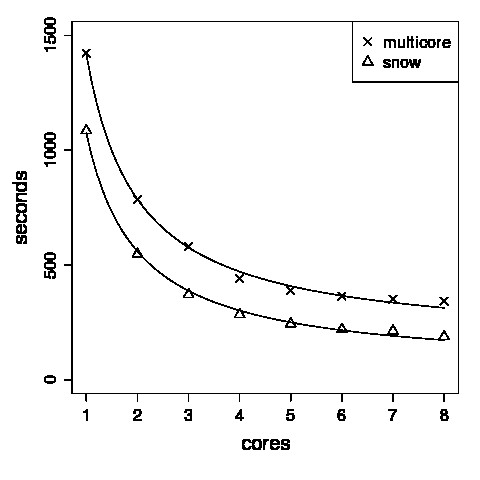
**Timings**. We tested the routine simc for breaking individual contacts and computing the Frobenius norm of the covariance matrix with respect to the original matrix using different numbers of cores. We compared the speed-up of the parallelization provided by the R packages multicore [31] and snow[18]. The elapsed time for both is fitted to a scaling law of the form *t*_CPU _≈ *a *+ *c *(#cores)^-1 ^with some unimportant constants *a*, *c *and *t*_CPU _the total CPU time, and #cores the number of multi-cores used.

We tested the efficiency of the code for this particular example using different numbers of cores (see Figure 4) in the parallelization provided by the snow package. Figure 4 suggests efficient parallelization up to 8 cores in accordance with Amdahl's law [27].

**Figure 5:**
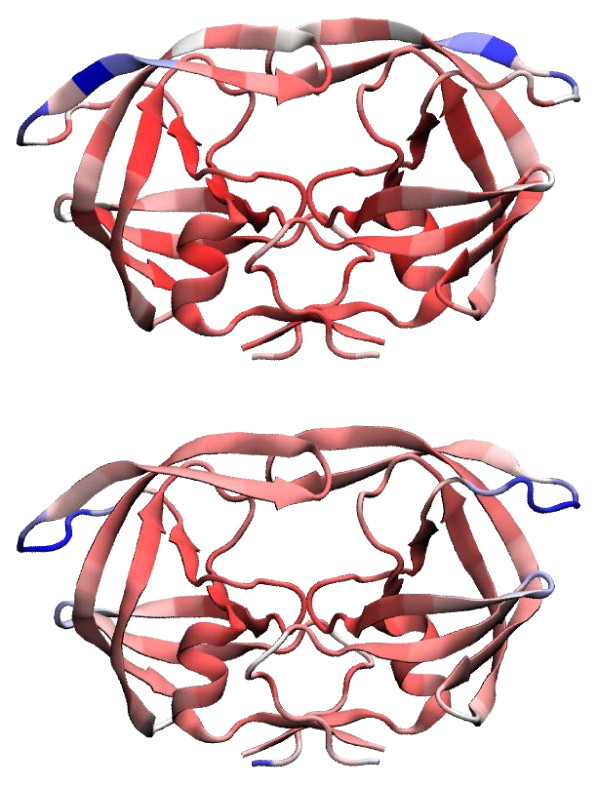
**Temperature factors**. B-factors for the HIV-1 protease [PDB:1KZK] computed using the ANM (top) and the SCPCP (bottom) model. The picture was rendered using VMD [32], "blue" indicates high, "white" intermediate and "red" low values. High temperature factors imply exibility and low ones rigidity.

Furthermore we determined the temperature factors (or B-factors), using the ANM and the SCPCP. The results are shown in Figure 5. As can be concluded from this graphic, the ANM detects larger flexible regions than the SCPCP.

### Future Trends & Intended Use

R[15] is a widely used and powerful environment for interactive analysis of statistical data in bioinformatics offering lots of additional software packages (e.g. from the Bioconductor[28] software project). We implemented the BioPhysConnectoR package in R to make the routines and underlying concepts accessible to a wide community allowing fast and parallelized network-based analysis of protein structures. Work is in progress to develop more efficient algorithms to compute covariance matrices for mutated systems and for biomolecular design [29] in the elastic network framework.

## Conclusions

In the BioPhysConnectoR package we provide routines to compare an original protein system to subsequently altered ones with mutated amino acid sequences or "broken" non-covalent contacts. Using sequence alignments we are able to score sequence changes and coevolution by the bio-mechanical ramifications of these changes. We can then use the biophysical modeling to annotate signals of coevolution in the sequence data. We include several options to alter the protocol of [13]: I) parametrization of bonds and contacts can be changed; II) including the well-known MJ and KE weighting scheme [22,23]; individual interactions in the structure can be altered; III) details on how to analyze mechanical changes can be modified by computing FNs just for subsets of residues; IV) dynamical and thermodynamical properties can be computed. Changes in the molecular mechanics for different scenarios (including mutations) can then be computed e.g. by the FN of the respective covariance matrices. The evolutionary connection of residues (indicated by high MI values) can be annotated by biophysical properties of the encoded molecule. In addition, a thermodynamical, reduced model is included to correlate the variability of protein sequences and thermodynamical implications. The package can furthermore be combined with state of the art optimization schemes to design molecules [29,30].

## Availability and requirements

**Project name: **BioPhysConnectoR

**Project home page: **http://bioserver.bio.tu-darmstadt.de/software/BioPhysConnectoR and CRAN at http://cran.r-project.org/

**Operating system: **cross-platform

**Programming language: **R and C/C++

**Requirements: **The R packages snow and matrixcalc are automatically installed from the CRAN repository.

**License: **GPL 2 license

**Any restrictions to use by non-academics: **none

## Authors' contributions

KH supplied the protocol for connecting sequence information and biophysical properties as well as for computing the SCPCP. The R implementation (including the C/C++ code) was carried out by FH and in parts by PW. All authors participated in writing the manuscript. All authors read and approved the final version of the manuscript.
